# Corticotropin releasing hormone as an identifier of bronchiolitis obliterans syndrome

**DOI:** 10.1038/s41598-022-12546-1

**Published:** 2022-05-19

**Authors:** Anna Niroomand, Haider Ghaidan, Oskar Hallgren, Lennart Hansson, Hillevi Larsson, Darcy Wagner, Martina Mackova, Kieran Halloran, Snejana Hyllén, Sandra Lindstedt

**Affiliations:** 1 Rutgers Robert Wood Johnson Medical School, New Brunswick, NJ USA; 2 Wallenberg Center for Molecular Medicine, Lund University, Lund, Sweden; 3 Department of Clinical Sciences, Lund University, Lund, Sweden; 4 Lund Stem Cell Center, Lund University, Lund, Sweden; 5 Department of Cardiothoracic Surgery and Transplantation, Skåne University Hospital, 221 85 Lund, Sweden; 6 Department of Pulmonology and Transplantation, Skåne University Hospital, Lund, Sweden; 7 Department of Experimental Medical Sciences, Lung Bioengineering and Regeneration, Lund University, Lund, Sweden; 8 Department of Medicine, University of Alberta, Edmonton, Canada; 9 Alberta Transplant Applied Genomics Center, University of Alberta, Edmonton, Canada; 10 Department of Cardiothoracic Anaesthesia and Intensive Care, Skåne University Hospital, Lund, Sweden

**Keywords:** Biomarkers, Immunological disorders, Medical research

## Abstract

Lung transplantion (LTx) recipients have low long-term survival and a high incidence of bronchiolitis obliterans syndrome (BOS), an inflammation of the small airways in chronic rejection of a lung allograft. There is great clinical need for a minimally invasive biomarker of BOS. Here, 644 different proteins were analyzed to detect biomarkers that distinguish BOS grade 0 from grades 1–3. The plasma of 46 double lung transplant patients was analyzed for proteins using a high-component, multiplex immunoassay that enables analysis of protein biomarkers. Proximity Extension Assay (PEA) consists of antibody probe pairs which bind to targets. The resulting polymerase chain reaction (PCR) reporter sequence can be quantified by real-time PCR. Samples were collected at baseline and 1-year post transplantation. Enzyme-linked immunosorbent assay (ELISA) was used to validate the findings of the PEA analysis across both time points and microarray datasets from other lung transplantation centers demonstrated the same findings. Significant decreases in the plasma protein levels of CRH, FERC2, IL-20RA, TNFB, and IGSF3 and an increase in MMP-9 and CTSL1 were seen in patients who developed BOS compared to those who did not. In this study, CRH is presented as a novel potential biomarker in the progression of disease because of its decreased levels in patients across all BOS grades. Additionally, biomarkers involving the remodeling of the extracellular matrix (ECM), such as MMP-9 and CTSL1, were increased in BOS patients.

## Introduction

Lung transplant patients face poor survival rates in comparison with other solid organ transplantations primarily due to a high incidence of chronic lung allograft dysfunction (CLAD), especially bronchiolitis obliterans syndrome (BOS)^1^. This is a major limitation to the long-term success of lung transplantation (LTx)^2–6^. The development of BOS is rare in the first year after LTx, but the rate increases quickly thereafter, with cumulative incidence rates as high as 40% to 80% within the first five years^2–4,6^. While several therapies have been attempted, they are generally ineffective. Bronchiolitis obliterans syndrome (BOS) results from an inflammation of the small airways in which attempted repair of injured epithelial cells leads to fibrotic narrowing^7^. As fibrotic obliteration of the lumen progresses, irreversible allograft injury and ultimately impaired gas exchange and allograft failure occurs^3,7^.

Clinical determination of BOS requires a permanent 20% drop in the forced expiratory volume in 1 s (FEV1) not attributable to a concurrent process, such as infection^8^. The difficulty of distinguishing between BOS and other diseases affecting pulmonary function can delay its diagnosis. Furthermore, as airway remodeling in BOS pathology is heterogenous, the histopathological analysis of lung biopsies has low sensitivity. Thus a need for a reliable set of biomarkers exists. Potentially through the use of a combination of parameters, biomarkers could diagnose or predict CLAD early in its process.

Clinical practice has so far focused on donor-specific antibodies as biomarkers, however they are not fully vetted in their ability to predict CLAD accurately^9–12^. Determining the development of CLAD as the disease becomes increasingly severe is imperative given the irreversible nature of the disease. Therefore, if biomarkers were accessible to clinicians, they would prove to be invaluable in the detection of allograft damage to provide an opportunity for intervention as disease progresses.

Proteomics, a field marked by recent technological developments, has been utilized in the search for biomarkers across a number of fields, including lung cancer and transplantation^13–15^. We hypothesized that a highly sensitive antibody-based proximity extension assay (PEA) could be used to distinguish between patients with and without BOS. Furthermore, we hypothesized that the assay could distinguish between BOS grades.

## Methods

### Samples

Patients who underwent double lung transplant (DLTx), who were in stable condition without ongoing infections and who were ≥ 2 years from transplant were selected. Patients who received a transplant from 1992 to 2016 were included within the present study. Those with restrictive allograft syndrome (RAS) were excluded for reasons of low sample size and limiting the focus of biomarker analysis to one form of pathophysiology as various forms of CLAD may have unique biomarkers. Eligible patients (n = 46) were phenotyped and assigned BOS grades based on pulmonary function tests, chest imaging, and transbronchial biopsies according to the International Society for Heart and Lung Transplantation (ISHLT) guidelines, with patient characteristics shown in Table 1^8,16^. According to this guideline, BOS grades are determined according to the decline of forced expiratory volume in one second (FEV_1_) relative to the patient’s baseline FEV_1_. A BOS grade of 0 equates to FEV of at least 90% of baseline, grade 1 as 66–80%, grade 2 as 51–65%, and grade 3 as at or less than 50%^13^. Spirometry measures should be combined with evaluation including imaging and bronchoscopy to identify and rule out other specific causes. Patients in this study were clinically diagnosed with BOS according to the guidelines of the ISHLT statement. Among 46 patients, 27 samples came from BOS grade 0, 8 from grade 1, 6 from grade 2 and 5 from grade 3. The major indications for transplantation included chronic obstructive pulmonary disease (n = 5 ), cystic fibrosis (n = 11), α1-antitrypsin deficiency (n = 9), pulmonary fibrosis (n = 7), pulmonary hypertension (n = 5), and “other” (n = 9), which included bronchiectasis, sarcoidosis, and graft-vs-host disease (Table 1). Plasma samples were collected at the time of enrollment in the study from patients at least 2 years following transplantation in stable condition with no known infection or progression of disease state. Baseline samples were then followed by another sample one year later. All samples were collected in EDTA tubes, centrifuged and kept frozen at -80 degrees C.

**Table 1:** Patient characteristics (n = 46).

**Variable**	
Sex; Female	28 (58%)
Age at Ltx, years	55 (21–73)
BMI, kg/m^2^	21 ± 4.8
**Diagnosis**	
COPD	5 (10.4%)
Cystic Fibrosis	11 (22.9%)
AAT1	9 (18.8%)
PF	7 (14.6%)
PH	5 (10.4%)
Other	9 (18.8%)
BOS Grade 0	27 (58.7%)
BOS Grade 1	8 (17.4%)
BOS Grade 2	6 (13.0%)
BOS Grade 3	5 (10.9%)
**Antirejection therapy**	
Tacrolimus	25 (54.3%)
Cyclosporine	20 (43.5%)
Everolimus	11 (23.9%)
Mycophenolate mofetil (MMF)	35 (76.1%)
Azathioprine	8 (17.4%)
Corticosteroids	41 (89.1%)
**FEV1 at baseline timepoint**	
BOS Grade 0	2.53 ± 0.68
BOS Grade 1	2.29 ± 0.49
BOS Grade 2–3	1.3 ± 0.55
**TLC at baseline timepoint**	
BOS Grade 0	5.69 ± 1.16
BOS Grade 1	5.38 ± 0.88
BOS Grade 2–3	5.78 ± 2.00
**FEV1 at 1 year follow-up**	
BOS Grade 0	2.50 ± 0.71
BOS Grade 1	2.03 ± 0.59
BOS Grade 2–3	1.38 ± 0.44
**TLC at 1 year follow-up**	
BOS Grade 0	5.68 ± 1.35
BOS Grade 1	4.93 ± 0.96
BOS Grade 2–3	5.11 ± 0.99

Eligible patients included those in stable condition, without ongoing infection, who were ≥ 2 years and did not have RAS. Numbers are expressed as the mean $x$ ± SD when parametric, median $x$ (range) when nonparametric or numerical values (%). Ltx = Lung transplantation; BMI = Body Mass Index; COPD = Chronic obstructive pulmonary disease; A1ATD = α-1-antitrypsin deficiency; PF = Pulmonary fibrosis; PH = pulmonary hypertension; Other includes bronchiectasis, sarcoidosis, and graft-vs-host disease; BOS, bronchiolitis obliterans syndrome; FEV1 = forced expiratory volume in 1 s; TLC = total lung capacity.

### Ethical considerations

The study was performed in accordance with the Declaration of Helsinki and was approved by the Swedish Ethical Board (Dnr 2017/396). All patients gave written, informed consent before entering the study.

### Proximity extension assay

644 proteins in plasma were analyzed using Olink Multiplex cell regulation, inflammatory, immune response, organ damage, development, cardiovascular II, and cardiovascular III panels (Olink, Uppsala, Sweden, https://www.olink.com). Each panel contains 92 antibody probe pairs that bind target proteins in the sample. The panels were chosen on the basis of coverage for a wide array of potential targets related to cell regulation, inflammation, immune response, and organ damage. A proximity-dependent DNA polymerization event between a pair of oligonucleotide-labeled antibodies to the target protein leads to the formation of a PCR reporter sequence which is then quantified by real-time PCR^17,18^. Internal, extension, and detection controls monitored deviation, as described by the manufacturer (www.olink.com). Proteins with a call rate of less than 85%, meaning those targets where less than 85% of individuals had a measurable concentration above the limit of detection, were removed from further analysis on the basis of recommended intra-plate variation from the manufacturer. Normalized protein expression (NPX) was calculated by subtracting out an external inter-plate control. The values are set relative to a correction factor determined by Olink and generated on a log2 scale with background level at 0. Further information about the PEA along with information on data processing and normalization are available from the manufacturer (www.olink.com).

### Validation of protein expression findings

In order to validate the PEA results, CRH and MMP-9 in plasma were measured by ELISA kits according to manufacturer’s instructions: (CRH ELISA kit (OKEH00623), Aviva Systems Biology, San Diego, CA, US, Human MMP9 ELISA Kit (ab246539), Abcam, Cambridge, UK). The kits rely on standard sandwich enzyme-linked immunosorbent assay technology using specific antibodies. The optical densities of results were read at 450 nm. Sensitivity of the CRH and MMP9 assays were 4.9 pg/mL and 10 pg/mL respectively.

Plasma samples were taken at baseline following DLTx and of those 46 patients, 32 were analyzed again after 1 year. 6 patients were excluded due to re-transplantation secondary to BOS, another 5 died, and 3 were lost in follow up.

Microarray data from transbronchial biopsies was obtained from a study of 457 biopsies collected from consenting patients across 10 centers from the GEO dataset GSE150156.

From this set, gene expression microarrays were conducted according to previously described methods^19^. Histologic analysis was undergone at the respective participating center according to the local standards of care, which allowed for categorization of the patients as either having chronic lung allograft dysfunction (CLAD) or non-CLAD .

### Statistical analysis

Linear regression analysis was conducted on the PEA data using the three BOS groups (grade 0 vs grade 1 vs grade 2–3). ELISA data are presented as mean and SEM. Statistically significant differences were determined by Student’s t-test (normally distributed data) and by the Mann–Whitney test (non-parametric data). Analysis was performed using GraphPad Prism. Significance was defined as: p < 0.001 (***), p < 0.01 (**), p < 0.05 (*), and p > 0.05 (not significant), apart from PEA values where statistical significance was set at p < 0.01 to counteract multiple comparisons.

### Heatmap

Heatmaps were generated in R version 3.5.3 with the ‘pheatmap’ package following scaling, normalization and data reduction. Each protein was placed on the same logarithmic scale by setting each limit of detection to zero. NPX values below the limit were set to zero. The average scaled NPX value for each protein across BOS Grade 0 samples was subtracted from each scaled NPX value. Positive values indicate NPX values above the level of detection and increased in comparison to the average scaled value across BOS grade 0. For proteins on multiple panels, the average scaled value was used. Unsupervised hierarchical clustering was performed using Euclidean distances in the ‘pheatmap’ package.

## Results

### Heatmap

**Figure 1:**
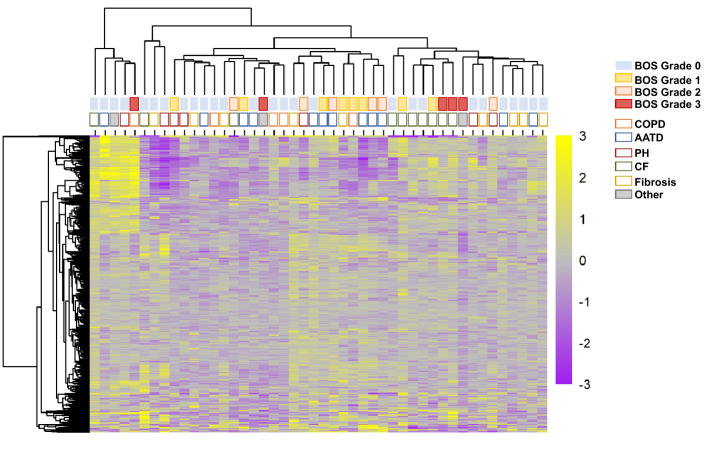
Heatmap of All Unique Proteins. Proteins detected using the proximity extension assay were placed on a logarithmic scale and NPX values were set to zero. Positive values indicate NPX levels above detection and increased compared to the average scaled NPX value for patients with BOS grade 0. Proteins detected in more than 15% of samples were included and then clustered using Euclidean distances. Assigned colors report both the BOS grade (top row) and well as the underlying diagnosis of each included patient (second row). COPD, chronic obstructive pulmonary disease; AATD, alpha-1 antitrypsin deficiency; PH, pulmonary hypertension; CF, cystic fibrosis; BOS, bronchiolitis obliterans.

Of 644 across all plates, 619 unique proteins were detected using PEA and 576 were retained after accounting for an 85% call rate. After allowing for clustering based on protein levels, BOS grades tended to group together and patients with cystic fibrosis grouped together (Fig. 1).

### PEA Proteomic analysis

#### Comparing BOS grade 0 to BOS grade 1–3

**Figure 2:**
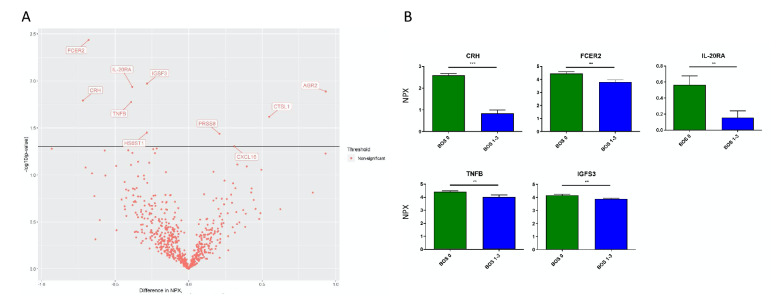
Detection of Biomarker Candidates Using Proximity Extension Assay. Patient samples were categorized as either a BOS grade 0 or a BOS grade 1–3. (**A**) Shows a volcano plot of the 644 proteins analysed using the proximity extension assay. A linear regression model compared the two groups with the solid line indicating a p value of 0.05. Proteins on the positive x-axis have higher NPX values in the BOS grade 1–3 group, and proteins on the negative x-axis have higher NPX values in the BOS grade 0 group. (**B**) Shows mean and SEM of five of the most significant proteins. These protein levels were all significantly lower among patients with BOS compared to those with grade 0. ** p < 0.01, *** p < 0.001. CRH, corticotropin releasing hormone; FCER2, low affinity immunoglobulin epsilon Fc receptor; IL-20RA, Interleukin-20 receptor subunit alpha; TNFB, TNF-β; IGFS3, immunoglobulin superfamily member 3; BOS, bronchiolitis obliterans syndrome.

Comparison of BOS grade 0 to grades 1–3 showed significant differences in plasma levels of: corticotropin releasing hormone (CRH), low affinity immunoglobulin epsilon Fc receptor (FCER2), Interleukin-20 receptor subunit alpha (IL-20RA), TNF-β (TNFB), and immunoglobulin superfamily member 3 (IGSF3). These proteins were significantly lower in patients who developed BOS (Fig. 2).

#### Comparing 3 groups: BOS grade 0 vs. BOS grade 1 vs. BOS grade 2–3

There were significantly lower levels of CRH, IL-20RA, and FCER2, both in patients who developed BOS grade 1 and in patients with BOS grades 2–3 compared to BOS grade 0.

**Figure 3:**
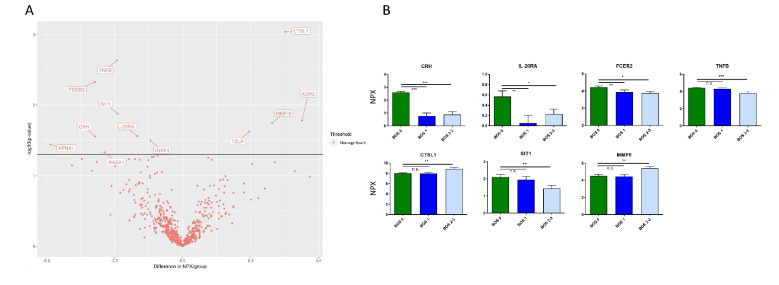
Comparison of BOS Grades 1 and 2–3 with Biomarker Candidates. Patient samples were subdivided between BOS grade 1 and grade 2–3 and then compared to BOS grade 0. Readouts are reported as normalized protein expression levels (NPX) relative to controls. (**A**) shows a volcano plot of the 644 proteins anlysed using Olink proteomics. A linear regression model was conducted with the solid line depicting a p-value = 0.05. The named proteins in the plot have a p-value < 0.05. Proteins on the positive x-axis have higher NPX values in the BOS grade 2–3 group, and proteins on the negative x-axis have higher NPX values in the BOS grade 0 group. (**B**) shows mean and SEM of seven of the most significant proteins. * *p* < 0.05, ** *p* < 0.01, *** *p* < 0.001. CRH, corticotropin releasing hormone; IL-20RA, Interleukin-20 receptor subunit alpha; FCER2, low affinity immunoglobulin epsilon Fc receptor; TNFB, TNF-β; CTSL1, cathepsin L1; SIT1, signaling threshold-regulating transmembrane adapter; MMP-9, matrix metalloproteinase 9; BOS, bronchiolitis obliterans syndrome.

Signaling threshold-regulating transmembrane adapter 1 (SIT1) and TNFB were significantly lower in BOS grades 2–3 compared to grade 0 but not in BOS grade 1 (Fig. 3).

**Figure 4:**
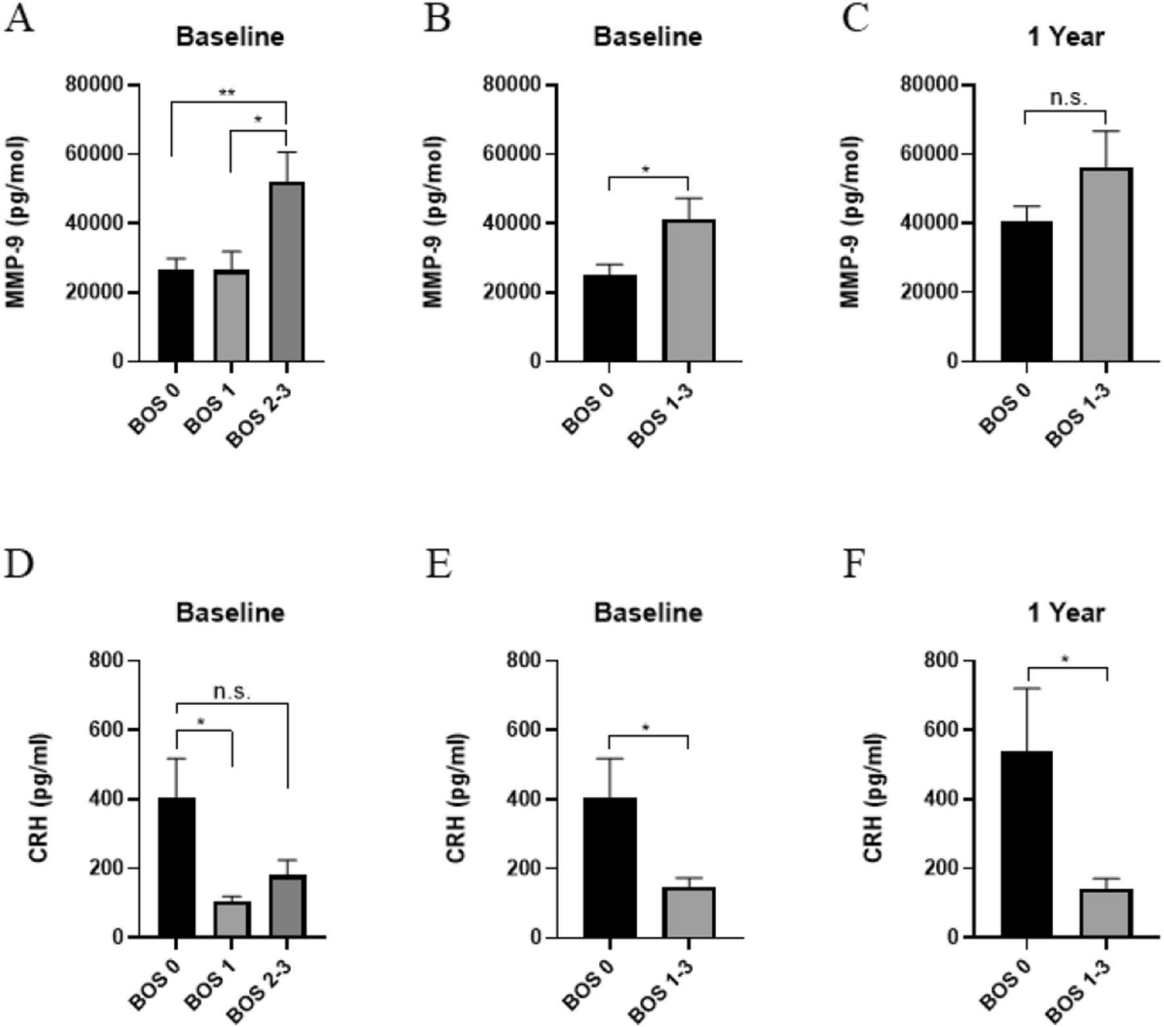
Expression of MMP-9 and CRH Across Timepoints. Using ELISA, MMP-9 and CRH were measured at baseline and analyzed using comparisons between BOS groups (**A**,**D**) or comparison of grouped BOS grades to grade 0 (**B**,**E**). Plasma concentrations were again measured at the 1 year follow up wherein grouped grades 1–3 were compared to BOS grade 0 (**C**,**F**). MMP-9 levels increased between grade 0 and BOS grades 1–3, with grade 2–3 exhibiting signifcantly higher levels than either of the two other grades. CRH levels were lower in BOS patients compared to grade 0, both at baseline and at the 1 year followup. * *p* < 0.05, ** *p* < 0.01. MMP-9, matrix metalloproteinase-9; CRH, corticotropin releasing hormone; BOS, bronchiolitis obliterans syndrome; ELISA, enzyme-linked immunosorbent assay.

Matrix metalloproteinase-9 (MMP-9), and cathepsin L1 (CTSL1) were significantly higher in patients with BOS grade 2–3 relative to grade 0 but not to BOS grade 1, which points toward higher levels of MMP-9 and CTSL1 in more advanced BOS stages (Fig. 4).

### Validation of PEA analysis

**Figure 5:**
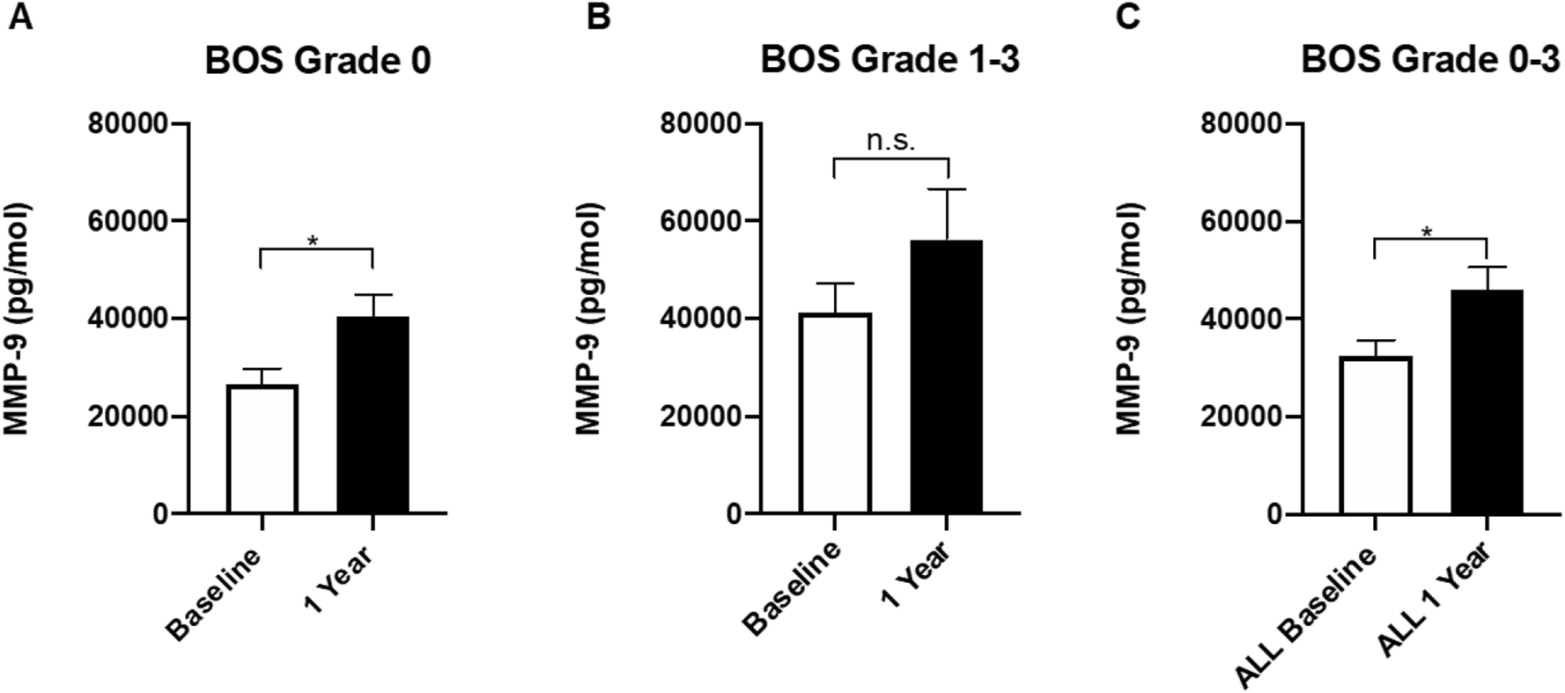
Elevation of MMP within BOS groups. Following patients from baseline to 1 year, MMP-9 levels in plasma increased within grades of BOS. This increase was statistically signifcant in BOS grade 0 (**A**) and across all BOS grades 0–3 grouped together (**C**). When BOS grades 1–3 were examined (**B**), a non-statistically significant increase can be appreciated. *p < 0.05. MMP-9, matrix metalloproteinase-9; BOS, bronchiolitis obliterans syndrome; ELISA, enzyme-linked immunosorbent assay.

In order to confirm the results of the proximity extension assay, a separate ELISA was conducted to measure protein levels using separate methodology. The baseline samples in 46 patients were also compared to the 32 patients who were able to be sampled at the one-year follow-up. At baseline, MMP-9 was significantly higher in those with BOS relative to those without. MMP-9 was significantly higher in grades 2–3 compared to either grade 1 or 0. After 1 year, there was no significant difference between BOS grade 0 and BOS grades 1–3, although there was a trend towards increased MMP-9 levels (Fig. 5).

At baseline, CRH was significantly lower in BOS grades 1–3 compared to grade 0. Only grade 1 showed a significant decrease in CRH plasma concentration compared to BOS grade 0. At the 1 year follow-up, CRH remained significantly decreased relative to grade 0.

**Figure 6:**
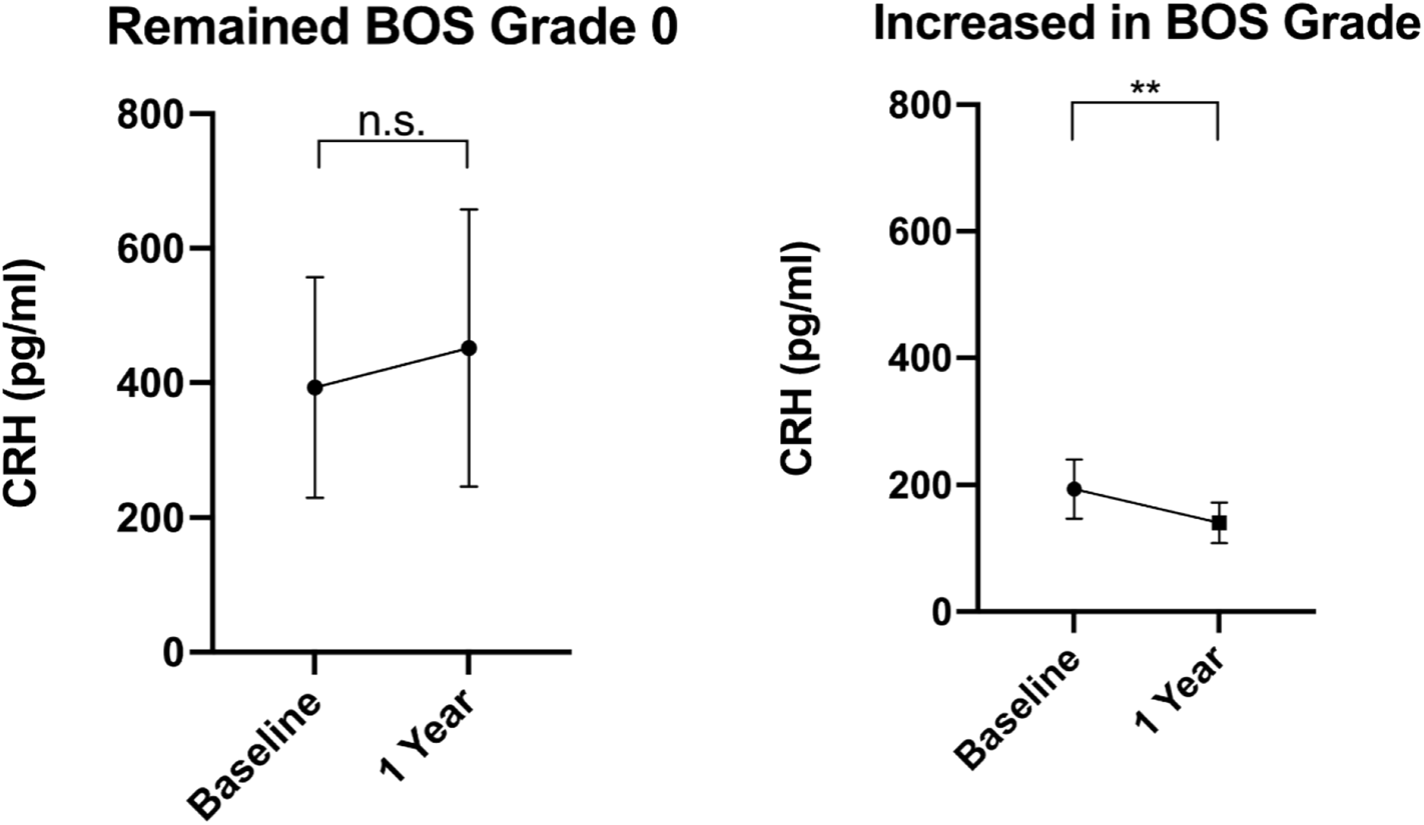
CRH Levels Tracked Through Patient Grade Changes. In comparing BOS grade 0 patients who maintained their status at the 1 year followup, later CRH levels are noted to not be statistically different from their baseline plasma concentrations (**A**). In patients who increased BOS grades after 1 year (**B**), CRH plasma levels were statistically lower in their second sample. **p < 0.01. CRH, corticotropin releasing hormone; BOS, bronchiolitis obliterans syndrome.

In examining patients who remained at BOS grade 0 from the baseline timepoint to the 1 year follow-up, there was no change in their CRH levels (Fig. 6). In patients whose BOS grade had increased, however, between these two timepoints, there was a significant decrease in CRH (Fig. 6).

From the microarray data gathered from samples across multiple centers in GSE150156, expression of CRH in CLAD was 21 compared to that of patients without CLAD who had an average value of 22 (p = 0.042, Table 2).

**Table 2:** Validation through external microarray data.

GEO ascension number	Participants	CRH expression		
		Non-CLAD	CLAD	
GSE150156	N = 457	22	21	*p* = 0.042

Values are expressed as quantile normalized intensity. CLAD, chronic lung allograft dysfunction; CRH, corticotropin releasing hormone; GEO, gene expression omnibus.

## Discussion

From the PEA analysis, there emerged patterns of grouping within patients as seen in the heatmap (Fig. 1). BOS grades grouped together, especially grades 0 and 1. When analyzed from the point of underlying diagnosis, a grouping of patients with cystic fibrosis is seen and alpha-1 antitrypsin deficiency patients showed a trend of grouping together though this was not observed to be a statistically significant correlation. This demonstrates the potential for further biomarkers for patients with these diseases or perhaps a protein profile characteristic of the disorder. Given that the cystic fibrosis grouping consisted of various BOS grades, further work could utilize this pattern of detected proteins to predict progression in rejection severity post transplantation.

This study demonstrated a drop in CRH levels as the grades of BOS increased (Figs. 2, 3, 4, 5). These changes were not found to correlate with anti-rejection therapy, change in therapy, patient characteristics, including diagnosis, age, or gender. The type of immunosuppression regimen was also not found to correlate with progression of BOS disease state within this study. The decreased levels of CRH across all BOS grades found using proximity extension assays was validated by ELISA at baseline and the 1-year follow-up which similarly found low CRH levels (Fig. 4). In support of its potential as a biomarker, CRH was unchanged in individuals who maintained a BOS grade of 0 between their baseline **and follow-up after 1 year (Fig. 5A). In patients whose grade increased at follow-up, however, a significant drop in plasma concentration was observed (Fig. 5B). Importantly, there is no statistical difference between the baseline levels of CRH within the patients that will go on to stay stable versus those that will progress in their BOS grade (p = 0.406). This supports the hypothesis that CRH measurements have the potential to reflect an increasing risk for BOS.

CRH thus emerges as a novel biomarker by which to monitor BOS. As the decrease was seen across all grades, its loss may be implicated in both early and later stages of BOS development (Fig. 2, 3). CRH is known as a major integrator of endocrine, autonomic, and immune responses to stress^20–22^. Its most prominent role is as the hypothalamic regulator of adrenocorticotropic hormone (ACTH) secretion within the hypothalamic–pituitary–adrenal (HPA) axis. The release of ACTH in turn stimulates adrenal cortisol synthesis, an anti-inflammatory hormone^23^. By way of its regulation of glucocorticoids, CRH serves to mediate anti-inflammatory responses throughout the body. Locally produced CRH in peripheral tissues, including the lungs^24,25^, indicates a direct role in facilitating inflammatory responses. It is also known to be expressed by immune cells, including lymphocytes and neutrophils^24,26^.

The distal actions of CRH via cortisol are anti-inflammatory while the direct action of CRH in peripheral tissues is pro-inflammatory, revealing a dichotomous function. It has been shown to stimulate mast cell degranulation, T-lymphocyte proliferation, antibody production, natural killer cell activity, leukocyte chemotaxis, vascular permeability, and the expression of cytokines and reactive oxygen metabolites^21^. CRH has been linked to lung mechanical dysfunction, and a lack of CRH has been tied to an increase in allergen-induced airway inflammation in asthma^27^. In *S. pneumoniae*-infected mice, the inflammatory cellular response was curtailed by an intranasal dose of CRH, resulting in increased survival^28^. This suggests that CRH can function in the management of immune and inflammatory responses.

The offspring of corticotropin-releasing hormone-deficient (CRH-KO) mice, who are themselves deficient in glucocorticoids, exhibit abnormal pulmonary development and consequently, respiratory insufficiency. The resultant neonatal death can be prevented by prenatal administration of glucocorticoids^29^. In baboon fetal lung explants, CRH strongly stimulates surfactant phospholipid synthesis, making CRH a potent inducer of fetal lung cell differentiation^30^. Low levels of surfactant protein A are associated with BOS following lung transplantation^31^ and may therefore be associated with decreased CRH. Notably, CRH has not yet been explored for the role it may play in BOS development. Other mediators of inflammation have been singled out as potential biomarkers, including IL-1RA and a host of other interleukins^32,33^ but CRH is a novel candidate. In order to separately confirm the relationship of the decrease of CRH in BOS patients seen in this study, we used gene expression data from transbronchial biopsies of lung tissue. In a microarray of 457 biopsies, there was a difference between the higher non-CLAD CRH values and the lower CLAD values (22 vs 21, p = 0.042). This reinforces the conclusion drawn here that CRH has potential as a biomarker of chronic graft rejection. It must be stressed that there is need of validation of CRH as a biomarker in independent cohorts at other worldwide centers and at the protein level in plasma.

That CRH acts in inflammation regulation is of particular importance given the chronic inflammation underlying the pathophysiology of CLAD. Lung myofibroblasts show increased activity in BOS studies and extracellular matrix (ECM) is deposited in a pathological manner correlated with small airway remodeling^12^. Molecules implicated in lung fibrosis and ECM remodeling, such as proteases, should be explored as biomarkers. In this study, CTSL1, a human cysteine cathepsin, and MMP-9 were increased in BOS patients.

Cathepsins are regarded as ubiquitous household enzymes, primarily involved in the lysosomal recycling and degradation of proteins. Cathepsins are believed to play specific functions in lung homeostasis and pathophysiological events such as lung fibrosis and ECM remodeling. Cathepsin C inhibitors administered to murine recipients prior to LTX exhibit decreased rates of early primary graft dysfunction (PGD)^34^. Given the regulation cathepsin C has on the maturation of neutrophil serine proteases, which are linked to the ischemia–reperfusion injury underlying PGD, cathepsins may play a larger role in mediating allograft dysfunction^35^.

In this study, MMP-9 was elevated across BOS grades 1–3. MMP-9 has been implicated in the degradation of ECM in both normal physiology and in lung diseases such as BOS^36–39^. Primary bronchial epithelial cells (BECs) isolated from lung donor trachea or bronchi co-cultured with activated T cells have reported promotion the production of MMP-9^38^. In the same study, an examination of a cohort of LTx recipients showed a significant increase in MMP-9 12 months before a clinical diagnosis of chronic dysfunction^38^. The finding of elevated serum levels was similarly echoed by Kastelijn et al.^40^ and by Ramirez et al.’s discovery of high MMP-9 in bronchoalveolar lavage fluid from BOS patients^41^.

The confirmatory finding of elevated MMP-9 in serum samples reported here lends credence to its utility as a biomarker which could be tracked over time. This study outlines a significant increase in MMP-9 between BOS grades 1–3 compared to grade 0 measured by both multiplex and ELISA techniques (Fig. 2, 3). At baseline, MMP-9 in BOS grades 2–3 are significantly higher than either grade 0 or grade 1 (Fig. 4A). By the 1-year follow-up, the difference between these two groups is lost (Fig. 4B), which could be the result of increasing MMP-9 levels in grade 0 patients yet to clinically manifest in graft dysfunction. More timepoints should be explored in the future to determine if individuals with the highest MMP-9 levels in the BOS grade 0 subset at 1 year progress to higher BOS grades later. This is of particular interest as MMP-9 measurements rose significantly during the 1 year observation within the grade 0 group and when all groups were considered together (Fig. 6A,C). This suggests that MMP-9 may potentially set the stage for graft dysfunction through ECM remodeling.

Just as remodeling has been explored in the context of chronic dysfunction, aberrant matrix alterations are implicated in the disease progression of idiopathic pulmonary fibrosis (IPF). IPF is a fibroproliferative disease marked by persistent collagen production leading to fibrosis of the alveolar interstitium^42^. Cells expressing FcεRII (CD23), the low affinity Fc receptor which binds IgE, have been found in aggregates within lung biopsies from IPF patients^43^. Of the two IgE receptor types, FcεRII distinguishes itself based on a distinct expression pattern. Found on monocytes, B cells and dendritic cells, it also binds CD21 on B cell surfaces. The receptor enhances antigen uptake and may mediate allergic inflammation^44^. While the pathogenesis of BOS is not yet fully elucidated, humoral immunity is implicated by evidence of antibody-mediated rejection in transplant patients^45^. In this study, FcεRII was found to be decreased in all BOS grades compared to grade 0 using multiplex panel detection, which might be secondary to immunosuppressive treatment (Fig. 3).

In examining other receptors identified by the panels, levels of interleukin-20 receptor subunit alpha (IL-20RA) were noted for significantly decreased values across BOS patients (Fig. 2). This IL-20RA deficiency was seen in patients with BOS grades 1, 2 and 3, displaying correlation of early and late disease processes with IL-20RA deficiency (Fig. 3). IL-20RA is a subunit of two heterodimeric cytokine receptors. Together with IL-20RB, it functions as a receptor for IL-19, IL-20, and IL-24. IL-20RA with IL-10RB, however, is a receptor for IL-26. In a rat model of lesions histopathologically resembling BOS, a microarray with qPCR validation identified the upregulation of several interleukins, including IL-24^46^.

TNF-β (TNFB) mediates inflammation and has been surveyed for its involvement in graft rejection. This proposed process is supported by the finding that patients with allograft rejection have systemic TNF elevation in renal and liver transplants. As an inflammatory cytokine, it is hypothesized to induce NFκB and MAPK signaling along with a host of other cytokines and chemokines. However, TNF is known to be responsible for both cellular survival and death^47^. In this study, a TNFB deficiency was seen not in patients with BOS grade 1 but in those with grades 2–3. Monitoring TNFB levels could thus be useful in predicting the progression to more severe BOS grades.

A significant decrease in IGSF3 was observed among patients with BOS grades 1–3 relative to grade 0 (Fig. 2). Composed of eight Ig-like C2-type (immunoglobulin-like) domains, IGSF3 has a high level of expression in the placenta, kidneys and lungs^48^.

Human bronchial epithelial cells with *IGSF3* knockdown have increased amounts of the sphingosine-1-phosphate which led to the conclusion that IGSF3 plays a role in cell adhesion^49^. IGSF3 knockdown was shown to impair structural cells leading to changes in cell morphology and reduced barrier function, implicating a potential role in lung tissue repair^49^. Gene expression of IGSF3 has been linked to the profile of four lung cancer subtypes, setting precedent for its potential as a biomarker^50,51^.

SIT1 is involved in biological processes such as signal transduction and the regulation of T cell activation. SIT1 additionally has a role in T cell homeostasis and the positive selection of T-cells^52–54^. In this study, while SIT1 was not decreased in BOS grade 1, it was significantly lower in patients with BOS grades 2–3 compared to patients without BOS development. Given the change in SIT1 seen in higher grades of BOS, its decrease may correlate with a later disease stage.

Limitations of this study included the relatively small sample size as well as the limited follow-up. Given the course of BOS and rates of survival following transplantation, a one-year follow-up was initiated as a starting point to begin to uncover potential differences and biomarkers that could occur in diseased and non-diseased patients. Further work could include a longer period in which to track the cohorts to determine relative changes in the proteins as patient health conditions were either maintained or deteriorated. In this study, microarray data collected from samples across ten centers supported the findings of lower CRH in the BOS patient group. The use of proximity extension assays to find the relative plasma concentrations of CRH in other patient cohorts at more centers would help support the findings of this current study.

## Conclusions

There is convincing evidence in the literature outlining the role of CRH in the modulation of inflammation as well as its tie to lung dysfunction. In the current study, decreases in CRH levels were observed in patients who developed BOS. These CRH deficiencies were not only remarked in patients with BOS grade 1 but also in patients with more severe grades 2 and 3. This reflects the importance of a CRH depletion across early and late processes of BOS development and helps to identify a potential marker as a novel diagnostic tool. Biomarkers involved in the remodeling of the ECM, such as MMP-9 and CTSL1, were also found in patients with severe BOS, in line with previous findings.

## Data Availability

The datasets used and/or analysed during the current study are available from the corresponding author on reasonable request.

## Abbreviations

BOS: Bronchiolitis obliterans
CTSL1: Cathepsin L1
CLAD: Chronic lung allograft dysfunction
CRH: Corticotropin releasing hormone
DLTx: Double lung transplant
ELISA: Enzyme-linked immunosorbent assay
FCER2: Low affinity immunoglobulin epsilon Fc receptor
FEV1: Forced expiratory volume in 1 s
IGSF3: Immunoglobulin superfamily member 3
IL-20RA: Interleukin-20 receptor subunit alpha
ISHLT: International Society for Heart and Lung Transplantation
LTx: Lung transplantation
MMP-9: Matrix metalloproteinase-9
NPX: Normalized protein expression
PEA: Proximity extension assay
RAS: Restrictive allograft syndrome
SIT1: Signaling threshold-regulating transmembrane adapter 1
TNFB: TNF-β

## References

[1] Vock DM, Durheim MT, Tsuang WM, Finlen Copeland CA, Tsiatis AA, Davidian M (2017). Survival benefit of lung transplantation in the modern era of lung allocation. Ann. Am. Thorac. Soc..

[2] Gauthier JM, Hachem RR, Kreisel D (2016). Update on chronic lung allograft dysfunction. Curr. Transplant. Rep..

[3] Kulkarni HS, Cherikh WS, Chambers DC, Garcia VC, Hachem RR, Kreisel D (2019). Bronchiolitis obliterans syndrome-free survival after lung transplantation: An International Society for Heart and Lung Transplantation Thoracic Transplant Registry analysis. J. Heart Lung Transpl..

[4] Verleden SE, Sacreas A, Vos R, Vanaudenaerde BM, Verleden GM (2016). Advances in understanding bronchiolitis obliterans after lung transplantation. Chest.

[5] Verleden SE, Vos R, Verleden GM (2019). Chronic lung allograft dysfunction: light at the end of the tunnel?. Curr. Opin. Organ Transpl..

[6] Verleden SE, Vasilescu DM, Willems S, Ruttens D, Vos R, Vandermeulen E (2014). The site and nature of airway obstruction after lung transplantation. Am. J. Respir. Crit. Care Med..

[7] Barker AF, Bergeron A, Rom WN, Hertz MI (2014). Obliterative bronchiolitis. N. Engl. J. Med..

[8] Meyer KC, Raghu G, Verleden GM, Corris PA, Aurora P, Wilson KC (2014). An international ISHLT/ATS/ERS clinical practice guideline: Diagnosis and management of bronchiolitis obliterans syndrome. Eur. Respir. J..

[9] Verleden SE, Vanaudenaerde BM, Emonds MP, Van Raemdonck DE, Neyrinck AP, Verleden GM (2017). Donor-specific and -nonspecific HLA antibodies and outcome post lung transplantation. Eur. Respir. J..

[10] Morrell MR, Pilewski JM, Gries CJ, Pipeling MR, Crespo MM, Ensor CR (2014). De novo donor-specific HLA antibodies are associated with early and high-grade bronchiolitis obliterans syndrome and death after lung transplantation. J. Heart Lung Transpl..

[11] Le Pavec J, Suberbielle C, Lamrani L, Feuillet S, Savale L, Dorfmuller P (2016). De-novo donor-specific anti-HLA antibodies 30 days after lung transplantation are associated with a worse outcome. J. Heart Lung Transpl..

[12] Tissot A, Danger R, Claustre J, Magnan A, Brouard S (2019). Early identification of chronic lung allograft dysfunction: The need of biomarkers. Front. Immunol..

[13] Bharti A, Ma PC, Salgia R (2007). Biomarker discovery in lung cancer–promises and challenges of clinical proteomics. Mass Spectrom. Rev..

[14] Lande JD, Patil J, Li N, Berryman TR, King RA, Hertz MI (2007). Novel insights into lung transplant rejection by microarray analysis. Proc. Am. Thorac. Soc..

[15] Indovina P, Marcelli E, Pentimalli F, Tanganelli P, Tarro G, Giordano A (2013). Mass spectrometry-based proteomics: The road to lung cancer biomarker discovery. Mass Spectrom. Rev..

[16] Verleden GM, Raghu G, Meyer KC, Glanville AR, Corris P (2014). A new classification system for chronic lung allograft dysfunction. J. Heart Lung Transpl..

[17] Assarsson E, Lundberg M, Holmquist G, Bjorkesten J, Thorsen SB, Ekman D (2014). Homogenous 96-plex PEA immunoassay exhibiting high sensitivity, specificity, and excellent scalability. PLoS ONE.

[18] Lundberg M, Eriksson A, Tran B, Assarsson E, Fredriksson S (2011). Homogeneous antibody-based proximity extension assays provide sensitive and specific detection of low-abundant proteins in human blood. Nucl. Acids Res..

[19] Halloran K, Parkes MD, Timofte I, Snell G, Westall G, Havlin J (2020). Molecular T-cellmediated rejection in transbronchial and mucosal lung transplant biopsies is associated with future risk of graft loss. J. Heart Lung Transpl..

[20] Chrousos GP (1995). The hypothalamic-pituitary-adrenal axis and immune-mediated inflammation. N. Engl. J. Med..

[21] Karalis K, Muglia LJ, Bae D, Hilderbrand H, Majzoub JA (1997). CRH and the immune system. J. Neuroimmunol..

[22] Webster EL, Torpy DJ, Elenkov IJ, Chrousos GP (1998). Corticotropin-releasing hormone and inflammation. Ann. N.Y. Acad. Sci..

[23] Vale W, Spiess J, Rivier C, Rivier J (1981). Characterization of a 41-residue ovine hypothalamic peptide that stimulates secretion of corticotropin and beta-endorphin. Science.

[24] Nezi, M., Mastorakos, G., & Mouslech, Z. Corticotropin releasing hormone and the immune/inflammatory response. In: Feingold, K. R., Anawalt, B., Boyce, A., Chrousos, G., de Herder, W. W., Dhatariya, K., et al., editors. Endotext. South Dartmouth (MA) (2000).

[25] Baigent SM (2001). Peripheral corticotropin-releasing hormone and urocortin in the control of the immune response. Peptides.

[26] Karalis K, Sano H, Redwine J, Listwak S, Wilder RL, Chrousos GP (1991). Autocrine or paracrine inflammatory actions of corticotropin-releasing hormone in vivo. Science.

[27] Silverman ES, Breault DT, Vallone J, Subramanian S, Yilmaz AD, Mathew S (2004). Corticotropin-releasing hormone deficiency increases allergen-induced airway inflammation in a mouse model of asthma. J. Allergy Clin. Immunol..

[28] Burnley B, Jones HP (2017). Corticotropin-releasing hormone improves survival in pneumococcal pneumonia by reducing pulmonary inflammation. Physiol. Rep..

[29] Muglia LJ, Bae DS, Brown TT, Vogt SK, Alvarez JG, Sunday ME (1999). Proliferation and differentiation defects during lung development in corticotropin-releasing hormone-deficient mice. Am. J. Respir. Cell Mol. Biol..

[30] Emanuel RL, Torday JS, Asokananthan N, Sunday ME (2000). Direct effects of corticotropin-releasing hormone and thyrotropin-releasing hormone on fetal lung explants. Peptides.

[31] Ericson PA, Mirgorodskaya E, Hammar OS, Viklund EA, Almstrand AR, Larsson PJ (2016). Low levels of exhaled surfactant protein a associated with BOS after lung transplantation. Transpl. Direct..

[32] Berastegui C, Roman J, Monforte V, Bravo C, Lopez-Meseguer M, Montero MA (2013). Biomarkers of pulmonary rejection. Transpl. Proc..

[33] Belperio JA, DiGiovine B, Keane MP, Burdick MD, Ying Xue Y, Ross DJ (2002). Interleukin-1 receptor antagonist as a biomarker for bronchiolitis obliterans syndrome in lung transplant recipients. Transplantation.

[34] Rehm SRT, Smirnova NF, Morrone C, Gotzfried J, Feuchtinger A, Pedersen J (2019). Premedication with a cathepsin C inhibitor alleviates early primary graft dysfunction in mouse recipients after lung transplantation. Sci. Rep..

[35] Lalmanach G, Saidi A, Marchand-Adam S, Lecaille F, Kasabova M (2015). Cysteine cathepsins and cystatins: from ancillary tasks to prominent status in lung diseases. Biol. Chem..

[36] Costa Fernandes CJD, Zambuzzi WF (2019). Fibroblast-secreted trophic factors contribute with ECM remodeling stimulus and upmodulate osteocyte gene markers in osteoblasts. Biochimie.

[37] Roda MA, Xu X, Abdalla TH, Sadik M, Szul T, Bratcher PE (2019). Proline-glycine-proline peptides are critical in the development of smoke-induced emphysema. Am. J. Respir. Cell Mol. Biol..

[38] Pain M, Royer PJ, Loy J, Girardeau A, Tissot A, Lacoste P (2017). T cells promote bronchial epithelial cell secretion of matrix metalloproteinase-9 via a C-C chemokine receptor type 2 pathway: Implications for chronic lung allograft dysfunction. Am. J. Transpl..

[39] Wu L, Luo Z, Zheng J, Yao P, Yuan Z, Lv X (2018). IL-33 can promote the process of pulmonary fibrosis by inducing the imbalance between MMP-9 and TIMP-1. Inflammation.

[40] Kastelijn EA, van Moorsel CH, Ruven HJ, Korthagen NM, Kwakkel-van Erp JM, van de Graaf EA (2013). YKL-40 and matrix metalloproteinases as potential biomarkers of inflammation and fibrosis in the development of bronchiolitis obliterans syndrome. Sarcoidos. Vasc. Diffuse Lung Dis..

[41] Ramirez AM, Nunley DR, Rojas M, Roman J (2008). Activation of tissue remodeling precedes obliterative bronchiolitis in lung transplant recipients. Biomark. Insights..

[42] Evans CM, Fingerlin TE, Schwarz MI, Lynch D, Kurche J, Warg L (2016). Idiopathic pulmonary fibrosis: A genetic disease that involves mucociliary dysfunction of the peripheral airways. Physiol. Rev..

[43] Pulkkinen V, Salmenkivi K, Kinnula VL, Sutinen E, Halme M, Hodgson U (2012). A novel screening method detects herpesviral DNA in the idiopathic pulmonary fibrosis lung. Ann. Med..

[44] Oettgen HC, Geha RS (1999). IgE in asthma and atopy: cellular and molecular connections. J. Clin. Invest..

[45] Todd JL, Palmer SM (2011). Bronchiolitis obliterans syndrome: the final frontier for lung transplantation. Chest.

[46] Morgan DL, Merrick BA, Gerrish KE, Stockton PS, Wang Y, Foley JF (2015). Gene expression in obliterative bronchiolitis-like lesions in 2,3-pentanedione-exposed rats. PLoS ONE.

[47] Ang RL, Ting AT (2019). Tumor necrosis factor-driven cell death in donor organ as a barrier to immunological tolerance. Curr. Opin. Organ Transpl..

[48] Saupe S, Roizes G, Peter M, Boyle S, Gardiner K, De Sario A (1998). Molecular cloning of a human cDNA IGSF3 encoding an immunoglobulin-like membrane protein: Expression and mapping to chromosome band 1p13. Genomics.

[49] Schweitzer KS, Jinawath N, Yonescu R, Ni K, Rush N, Charoensawan V (2020). IGSF3 mutation identified in patient with severe COPD alters cell function and motility. JCI Insight..

[50] Watanabe T, Miura T, Degawa Y, Fujita Y, Inoue M, Kawaguchi M (2010). Comparison of lung cancer cell lines representing four histopathological subtypes with gene expression profiling using quantitative real-time PCR. Cancer Cell Int..

[51] Zhang HH, Zhang ZY, Che CL, Mei YF, Shi YZ (2013). Array analysis for potential biomarker of gemcitabine identification in non-small cell lung cancer cell lines. Int. J. Clin. Exp. Pathol..

[52] Marie-Cardine A, Kirchgessner H, Bruyns E, Shevchenko A, Mann M, Autschbach F (1999). SHP2-interacting transmembrane adaptor protein (SIT), a novel disulfide-linked dimer regulating human T cell activation. J. Exp. Med..

[53] Posevitz V, Arndt B, Krieger T, Warnecke N, Schraven B, Simeoni L (2008). Regulation of T cell homeostasis by the transmembrane adaptor protein SIT. J. Immunol..

[54] Simeoni L, Posevitz V, Kolsch U, Meinert I, Bruyns E, Pfeffer K (2005). The transmembrane adapter protein SIT regulates thymic development and peripheral T-cell functions. Mol. Cell Biol..

